# Effects of Air Pollutant Exposure on Acute Myocardial Infarction, According to Gender

**DOI:** 10.5935/abc.20160117

**Published:** 2016-09

**Authors:** Tássia Soldi Tuan, Taís Siqueira Venâncio, Luiz Fernando Costa Nascimento

**Affiliations:** 1 Departamento de Medicina - Universidade de Taubaté,Taubaté, SP - Brazil; 2 Departamento de Energia - Universidade Estadual Paulista - Campus de Guaratinguetá,Guaratinguetá, SP - Brazil

**Keywords:** Myocardial Infarction, Environmental Pollutants, Gender Identity, Sulfur Dioxide, Carbon Monoxide

## Abstract

**Background:**

There is evidence of the effects of air pollution on hospital admissions due to cardiovascular diseases, including myocardial infarction.

**Objective:**

To estimate the association between exposure to air pollutants and hospital admissions due to myocardial infarction according to gender, between January 1^st^ 2012 and December 31^st^ 2013, in São Jose dos Campos-SP.

**Methods:**

An ecological time series study was carried out with daily data of admissions due to AMI, pollutants CO, O_3_, PM_10_, SO_2_, and NO_2_, according to gender. We used the Poisson regression generalized linear model to estimate the relative risks of hospital admissions with lags of 0-5 days, adjusted for temperature, humidity, seasonality and days of the week.

**Results:**

There were 1837 admissions for ischemic heart diseases, with 636 women and 1201 men. For females, the risks were significant for CO in lag 0 (RR = 1,09), lag1 (RR = 1,08) and lag 5 (RR = 1,10) and SO_2_ in lag 0 (RR = 1,10) and 3 (RR = 1,09). For men there was significance of the CO in, lag 3 and lag 5 (RR = 1,05). There was significance, regardless of gender, for CO at lag 1 (RR = 1,05) and lag 5 (RR = 1,07) and lag 0 for SO_2_ (RR = 1,06).

**Conclusion:**

The data presented show the important role of CO and SO_2_ in the genesis of myocardial infarction admissions, and responses to pollutant exposure are different if analyzed by gender and together - hence the importance of a stratified analyses.

## Introduction

Great evidence indicating that air pollution in our environment is enough to cause health damages, and the need to define regulatory process regarding air quality standards make it pivotal to better outline this association, identifying special population groups, specific pathologies, and environmental levels that lead to the exposure-disease process and death. Accordingly, information from systematic investigations with locally generated data are of great importance to subsidise planning and assessment of health care programs focused on this issue.^1^

Cardiovascular diseases are still the main cause of death in Brazil, accounting for almost 32% of all deaths. Moreover, it is the third leading cause of hospital admissions in the country. Among them, acute myocardial infarction is still one of the primary causes of morbidity and mortality. The study of acute myocardial infarction (AMI) is essential due to its high prevalence, morbidity and mortality. Epidemiological studies show general mortality rates of around 30%, with half of deaths occurring in the first two hours of the event, and 14% of patients dying before receiving medical treatment.^2^

In Brazil, in 2014, 95,000 hospital admissions for acute myocardial infarction were recorded; in the state of São Paulo, there were 27,000 (http://tabnet.datasus.gov.br/cgi/tabcgi.exe?sim/cnv/obt10uf.def).^3^

Studies from metropolitan areas and mid-sized cities have shown an association between admissions for AMI and exposure to air pollutants, with particulate matter (PM_10_), ozone (O_3_), sulphur dioxide (SO_2_), nitrogen dioxide (NO_2_), and carbon monoxide (CO) as the ones most highly associated to admissions for AMI.^1,4-16^

Carbon monoxide, which is still understudied, is released into the atmosphere by natural sources (volcanic activity, electrical discharges and natural gas emissions) and as a product of incomplete combustion of fossil fuels, heating systems, thermal coal plants, biomass and tobacco burning. Its significance lies in its affinity for hemoglobin, which is 240 greater than oxygen's.

Further epidemiological evidence that has been growing in different studies is the categorization by gender. Several studies show more pronounced effects in women than in men, but literature is still inconsistent in regards to that. Just as the outcome of hospital admission, there's also evidence of higher mortality in women in percutaneous coronary interventions.^5^

Several studies in areas such as The USA, Canada, and Europe show gender differences, varying according to age, in mortality from AMI and a higher risk of death in younger women compared to their male counterparts, and also different effects of risks in hospital admission due to respiratory diseases.^6-17^

The aim of this study is to estimate the association between exposure to air pollutants and hospital admissions for AMI (in individuals over 50 years of age), categorized by gender, between January 1^st^ 2012 and December 31^st^ 2013, in the city of São José dos Campos - SP.

## Methods

Ecological time series study with data relative to hospital admissions for AMI (ICD-10 from J20.0 to J24.0) in individuals of both genders, over 50 years of age, residents of São José dos Campos, SP. The study period was between January 1^st^, 2012 and December 31^st^, 2013. Admission data were obtained from the DATASUS portal.^3^ All actions carried out during the period of admission must be notified to the Brazilian Unified Health System (*Sistema Único de Saúde* - SUS) via a Hospital Admission Authorization (AIH), which is registered and filed, and payment to service providers for the procedures are made by SUS. Among the variables obtained in this portal, the ones used were relative to patients' gender, age (in years) and main diagnosis.

### Place of Study

São José dos Campos is a Brazilian municipality in the interior of the state of São Paulo, in the mesoregion of Vale do Paraíba Paulista, 84 km east of the capital of the state. It houses 650,000 people, and has a 130,000 vehicle fleet per day, of which only the minority are heavy (buses and trucks). It is an important economic center with companies in the fields of technology, education and research centers. Its geographical location is 23°11' S, 45°53'W.

Studied pollutants were PM_10_, SO_2_, O_3_, NO_2_ (*µ*g/m^3^) and CO (ppb), and values of PM_10_, SO_2,_ NO_2_ and CO were quantified by daily averages, and values for O_3_ were from a maximum of 8 hours. Such values were quantified by the Environmental Company of the State of São Paulo (CETESB),^18^ which relies on a measuring station in São José dos Campos, as well as information on minimum, mean, and maximum temperatures, relative air humidity, seasonality, and days of the week. From those data, minimum temperature and relative air humidity were used.

Hospital admission is a counting, discreet event for which the Poisson Regression is indicated to estimate the relative risks of exposure in the outcome - hospital admission. A data bank was built with daily admission data, for each pollutant and climatic variable. Lags of 0-5 days were considered because the effects of exposure to pollutants may be evidenced not only on the same day, but also days after exposure. Thus, a Poisson regression generalized linear model (GLM) was selected. Models with an isolated pollutant and with four pollutants simultaneously were built, adjusted by the minimum temperature, relative air humidity, seasonality, and days of the week. The analyses were carried out considering females, males, and both genders to identify possible differences in the relative risks for hospital admission for infarction, according to these strata. Pearson correlation values were obtained among the independent variables and presented in a table.

For the analysis, we used the software Stata V10. Coefficients provided by the Poisson Regression were transformed into relative risks (RR) with respective confidence intervals of 95%. In the case of significant association between exposure to a certain pollutant and hospital admission, we considered increases (AUM-RR) of 300 ppb for CO, and 2*µ*g/m^3^ for SO_2_ expressed in percentage points, according to the expression AUM-RR (%) = (exp^(***coef*** * AUM^-1)*100, in which *coef* is the numeric value of the coefficient provided by the Poisson Regression and AUM are the above values considered for CO and SO_2_. The variables were presented with the value of their means and respective standard deviations in a table.

Counsel from the Ethics Committee was waived since this is an ecological study and the data is publically available on the net, and also because of the impossibility to identify the subject of the analysis. The significance level adopted was of alpha = 5%.

## Results

A total of 1837 individuals were admitted for ischemic heart diseases, of which 636 (34.6%) were women, and 1201 (65.4%) were men. The mean concentration of the pollutants (*µ*g/m^3^), standard deviation, minimum and maximum are depicted in table 1.

**Table 1:** Descriptive analysis of study variables: Mean, standard deviation (sd), minimum and maximum values (Min – Max). São José dos Campos-BR, 2012-2013

**Variables**	**Mean (sd)**	**Min – Max**
Admissions	2.5 (1.8)	0.0 -- 9.0
PM_10_ (µg/m^3^)	22.6 (10.5)	6.0 -- 81.0
O_3_ (µg/m^3^)	74.1 (34.4)	1.0 -- 213.0
CO (ppb)	883 (459)	200 -- 3400
SO_2_ (µg/m^3^)	2.7 (2.3)	0.0 -- 29.0
NO_2_ (µg/m^3^)	48.2 (17.7)	11.0 -- 112.0
Humidity %	57.4 (16.4)	24.0 -- 99.0
Minimum temperature ºC	15.6 (3.5)	4.4 -- 21.9

Table 2 presents the correlation matrix between the study variables (environmental pollutants, climatic variables, and number of admissions) for both genders. Strong correlations between pollutants were observed, except for O_3_ and CO.

**Table 2:** Pearson correlation matrix between atmospheric variables for both genders. São José dos Campos, São Paulo, 2012/2013

	**Min. Temp.#**	**RH**	**PM_10_**	**SO_2_**	**CO**	**O_3_**	**NO_2_**
Min. Temp	1						
RH	0.143**	1					
PM_10_	-0.308**	-0.329**	1				
SO_2_	-0.058	-0.084*	0.167**	1			
CO	-0.288**	0.001	0.360**	0.099*	1		
O_3_	0.197**	-0.480**	0.149**	0.220**	0.037	1	
NO_2_	-0.410**	-0.169**	0.517**	0.128**	0.358**	0.171**	1

Min. Temp: minimum temperature; RH: relative air humidity.

* p-value r < 0.05

** p-value r < 0.01.

Exposure to pollutants, considering the increase in their concentrations of 300 ppb, was associated to CO in both genders in lags 1 (RR = 1.05) and 5 (RR = 1.07); in women in lags 0 (RR = 1.09), 1 (RR = 1.08), and 5 (RR = 1.10); and in men in lags 3 (RR = 1.06) and 5 (RR = 1.05). For SO_2_, the effects were observed in both genders in lag 0 (RR = 1.06); in women in lags 0 (RR = 1.10) and 3 (RR = 1.09); and no exposures with statistical significance were identified in men.

From the values obtained from the generalized linear model and its standard deviations, the confidence interval for the relative risk of admission for acute myocardial infarction was calculated.

**Figure 1:**
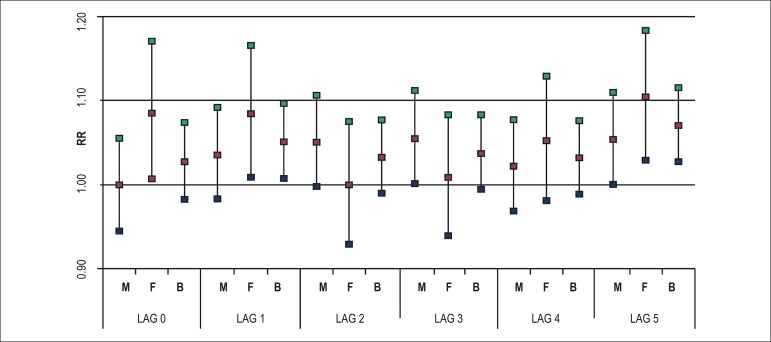
Relative risk for CO exposure according to lag 0 to lag 5 for males (M), females (F), and both genders (B). São José dos Campos. 2012-2013.

**Figure 2:**
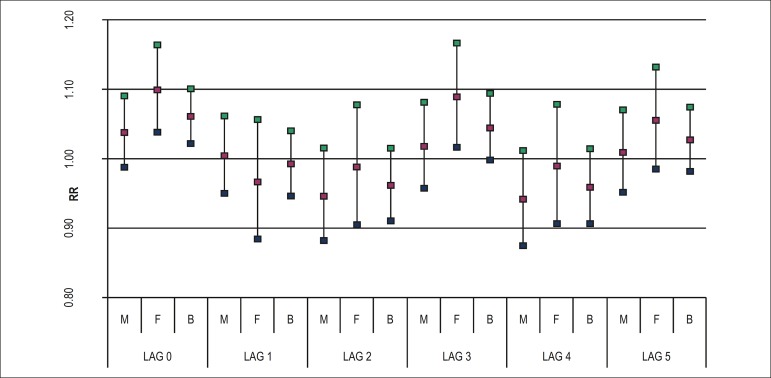
Relative risk for SO_2_ exposure according to lag 0 to lag 5 for males (M), females (F), and both genders (B). São José dos Campos. 2012-2013.

With an increase of 300 ppb for CO, relative risks and respective confidence intervals (CI 95%) are in Figure 1 for both genders, leading to an increase of 10 percentage points for women, and up to 7 points when both genders are analysed. In the case of SO_2_, as shown in Figure 2, the increase of 2,0 *µ*g/m^3^ implied an increase of up to 5 percentage points for both genders, up to 100 pp for women, and non-significant for men.

## Discussion

This study, to the best of our knowledge, is the first to analyse stratified exposure by gender - it identified the importance of exposure to CO and SO_2_ in the genesis of hospital admissions for ischemic heart diseases, in individuals over 50 years old, in the city of São José dos Campos, separating the individuals by gender. For CO, the effects were evident 1 and 5 days after exposure when both genders were analysed; stratifying admissions, they occurred on the same day, 1, and 5 days after exposure for women, and 3 and 5 days after exposure for men. In relation to SO_2_, effects were evident in lag 0 for both genders, lag 0 and 3 for women, and with no statistical difference for men.

Considering this is a multipollutant model, other pollutants were analysed, but no association was found for them.

The categorization by gender has been approached in several studies, showing epidemiological significance, but there are still no studies with a biological explanation.

According to Clougherty^17^, several studies suggest that the response to air pollutant exposure differs for men and women, or for boys and girls. The explanation, however, is still unclear, while modifications are observed as a result of biological differences linked to gender (e.g., hormones and body size) or gender differences in activity patterns, coexposure, or exposure quantification accuracy. Numerous modifications consist of the modification of these two factors (exposure pattern and biological response).

An association between exposure to SO_2_ and cardiovascular diseases, especially deaths by stroke, was shown in São José dos Campos, associated to ozone pollutants and particulate matter, in individuals over 50 years of age. SO_2_ concentrations were 4 *µ*g/m^3^.^7^ In a study in São Paulo, exposure to SO_2_ was associated to admissions for circulatory and ischemic heart diseases,^1^ as the effects were similar to the CO exposure, but with greater intensity. Sunyer et al. have also shown the association of SO_2_ exposure and cardiovascular diseases in seven European cities. Concentrations of the pollutant were between 5 and 21 *µ*g/m^3^, and increases of 10 *µ*g/m^3^, in these concentrations, implicated in significant increases between 0.7% and 1.2% in the number of hospital admissions for cardiovascular diseases, especially ischemic heart diseases.^8^ Atkinson et al.^9^ showed the significant effect of SO_2_ exposure in isolation and adjusted by gender, age, smoking habit, BMI, and comorbidities such as diabetes and arterial hypertension in hospital admissions for acute myocardial infarction, stroke, arrhythmias and heart failure.

SO_2_ exposure was also significant in admissions for CVD with or without diabetes.^10^ Our study also showed that increases of 2 *µ*g/m^3^ in SO_2_ concentrations, adjusted by concentration of other pollutants, implicate in a significant increase in risk for women (RR = 1.10), which contributed to high risk rates in the absence of stratification by gender. That is, when assessed for both genders (RR = 1.06) because the effects of exposure were not significant for men.

In this study, CO exposure, in São José dos Campos, was also significant for admissions for AMI. Such findings are in accordance with those found by Gouveia et al.,^1^ when concentrations were 3240 ppb, reaching a maximum of 12600 ppb, values far above the ones observed in São José dos Campos, which had a mean of 883 ppb, and a maximum of 3400 ppb. Risks observed in São Paulo, according to an increase of 1000 ppb in CO concentrations were RR = 1.016, and the most significant discrepancy was a moving average of 2 days. CO exposure was significant to emergency services for cardiovascular diseases with or without diabetes. This association was more evident on the same day as exposure (lag 0) in non-diabetic individuals.^10^

In a study done in Chicago, CO effect on admissions for heart failure was dependent on temperature, with the magnitude of the effect increasing as the temperature dropped.^11^ Carbon monoxide concentration recorded on the day of admissions showed, among the pollutants, the strongest and most consistent association with hospital admission rates, simultaneously adjusting to temperature, dew point, and other air pollutants, for a change of 1000 ppb to 3000 ppb, interquartile interval, and relative risk of RR = 1.065 (CI 95% = 1.028-1.104).^12^

A study developed in China analysed a sample of patients with acute myocardial infarction in several Chinese hospitals in 2001, 2006, and 2011, and showed that in-hospital mortality rate was higher among women than men (17.2% vs 9.1%; p < 0.01; OR 2.07; 95% IC 1.85-2.33). Odds ratio not adjusted for mortality in women, in comparison to men, was of 2.20 (95% CI 1.59-3.04); 2.21 (95% CI 1.74-2.79); 1.37 (95% CI 1.15-1.65); and 1.25 (CI 95% 0.97-1.63) for the ages <60; 60-69; 70-79 and ≥ 80 years, respectively. After adjusting to the characteristic of patients, hospital and year of study, OR for mortality, comparing men and women, was 1.69 (95% CI 1.01-2.83); 1.64 (95% CI 1.24-2.19); 1.15 (CI 95% 0.90-1.46); and 0.82 (95% CI 0.60-1.11) for the ages <60; 60-69; 70-79; and ≥ 80 years, respectively. Gender-age interaction for mortality was statistically significant (p = 0.009), even after adjustment for a wide range of confounders, and did not vary over time or in rural/urban areas.^6^

The associations between SO_2_ and CO and admissions for AMI are in keeping with the findings of Koken et al.^13^ associating SO_2_ to an increase in hospital admissions for cardiac arrhythmias, and CO significantly associated to admissions for congestive heart failure. Additionally, they found more hospital admissions for cardiovascular diseases in men than in women.

A study done in São Paulo found greater effects of air pollution on congestive heart failure in men, and on cardiovascular and ischemic heart diseases in women. This reinforces the need for additional studies focusing on the modification of air pollution effects on health by gender.^14^ Kan et al.^15^ showed that effects of air pollutant exposure, SO_2_ amongst them, were more evident in women. An increased risk of death by stroke was found in older women after PM_10_ exposure.^15,16^

On the other hand, Cakmak et al.^19^ did not find a significant association between cardiac disease and air pollution that was influenced by gender. In another study, according to a gender stratified analysis, no statistically significant difference was found between pollutants and mortality from cardiovascular diseases (CVD) in women, and among men, only NO_2_ was significantly associated to mortality from CVD.^20^ Zeka et al.^21^ identified a smaller effect of exposure to PM10 in mortality from cardiovascular diseases in women over 60 than in men in the same age group. A possible explanation for that would be hormonal. In post-menopausal women (over 60), with PM_10_ exposure, mortality risk from cardiac diseases was five times higher than in pre-menopausal women. However, men in the same age groups, presented a risk two times higher in the over 60 group.^22^ A study developed in Shanghai about the role of air pollutants in daily mortality showed that SO_2_ and NO_2_ exposure effects in mortality were slightly higher in women than in men. The mean concentration of SO_2_ was 45 *µ*g/m^3^ and of NO2, 67 *µ*g/m^3^; carbon monoxide was not included in this study.^15^

Chen et al.^23^ found risk of death from coronary disease (CD), stemming from PM_10_ and PM_2.5_ exposure, that was significantly higher in women than in men, in the analysis of a single pollutant as well as in the multipollutant analysis. SO_2_ exposure was not associated to death from CD. A reason for such finding would be that PM_10_ and PM_2.5_ deposition is more localized and more intense in women than in men - the smaller number of red cells in women might make them more sensitive to the toxic effects of air pollutants.^23^

In a study with over 65,000 post-menopausal women, exposure to fine particulates was associated to the incidence of cardiovascular disease and death.

### Limitations

This study may have limitations, among which are the own limitations of ecological studies. It is not possible to point out the causality between exposure and outcomes other than to point out associations between exposure and outcomes. It is not possible to identify if the admitted individual was exposed and if the exposed individual was admitted. There may error in diagnoses recorded on Datasus, leading to sub-notations and over-notations in cases of infarction. However, Datasus is an official, reliable and widely used source in the areas of air pollutant exposure effects and illness. We also did not include hospital admissions through health plans or health insurance. It is worth noting that Datasus does not contemplate information on factors or comorbidities associated to ischemic heart diseases such as smoking, overweightness and obesity, hypercholesterolaemia, and previous diseases of the circulatory system. Concentrations were considered homogenous in the entire city, and it was assumed that exposures happened homogeneously and that people had free movement around the city.

Despite these limitations, other than pointing out the risks of air pollutant exposure in the genesis of admissions for myocardial infarction in a mid-sized city, the importance of the study lies in the fact that there are differences in the responses to pollutant exposure according to gender, and that analyses involving air pollutant exposure and circulatory diseases stratified by gender must be adopted.

## Conclusions

This study revealed that the global impact assessment of air pollution on health, through time series studies, is important to strengthen the implementation of environmental health surveillance by the health sector. The results show the direct estimate of population illness due to a variation of atmospheric pollutant concentrations. It is suggested that preventive and educational measures, through the media, keep the population informed about environmental pollution conditions, as well as the optimal places for leisure and sports.
